# Smart Design of Mitochondria-Targeted and ROS-Responsive CPI-613 Delivery Nanoplatform for Bioenergetic Pancreatic Cancer Therapy

**DOI:** 10.3390/nano11112875

**Published:** 2021-10-28

**Authors:** Yi-Mei Zhang, Meng Xia, Rui Ao, Li-Xia Gao, Yan Tang, Jiu-Hong Huang, Ya-Fei Luo, Zhong-Zhu Chen, Bo-Chu Wang, Zheng Huang

**Affiliations:** 1 National & Local Joint Engineering Research Center of Targeted and Innovative Therapeutics, Chongqing Key Laboratory of Kinase Modulators as Innovative Medicine, College of Pharmacy & International Academy of Targeted Therapeutics and Innovation, Chongqing University of Arts and Sciences, Chongqing 402160, China; yimeizhang@cqwu.edu.cn (Y.-M.Z.); xm269691792@163.com (M.X.); aor1027@163.com (R.A.); LixiaGao@cqwu.edu.cn (L.-X.G.); 20160063@cqwu.edu.cn (Y.T.); jhhuang@cqwu.edu.cn (J.-H.H.); luoyf@cqwu.edu.cn (Y.-F.L.); 18883138277@163.com (Z.-Z.C.); 2 Key Laboratory of Bio-Theological Science and Technology of Ministry of Education, College of Bioengineering, Chongqing University, Chongqing 400045, China; wangbc2000@126.com

**Keywords:** mitochondria-targeting, ROS-responsive, drug delivery, nanoparticles, pancreatic cancer therapy, CPI-613

## Abstract

Mitochondria, as the powerhouse of most cells, are not only responsible for the generation of adenosine triphosphate (ATP) but also play a decisive role in the regulation of apoptotic cell death, especially of cancer cells. Safe potential delivery systems which can achieve organelle-targeted therapy are urgently required. In this study, for effective pancreatic cancer therapy, a novel mitochondria-targeted and ROS-triggered drug delivery nanoplatform was developed from the TPP-TK-CPI-613 (**TTCI**) prodrug, in which the ROS-cleave thioketal functions as a linker connecting mitochondrial targeting ligand TPP and anti-mitochondrial metabolism agent CPI-613. DSPE-PEG2000 was added as an assistant component to increase accumulation in the tumor via the EPR effect. This new nanoplatform showed effective mitochondrial targeting, ROS-cleaving capability, and robust therapeutic performances. With active mitochondrial targeting, the formulated nanoparticles (**TTCI** NPs) demonstrate much higher accumulation in mitochondria, facilitating the targeted delivery of CPI-613 to its acting site. The results of in vitro antitumor activity and cell apoptosis revealed that the IC_50_ values of **TTCI** NPs in three types of pancreatic cancer cells were around 20~30 µM, which was far lower than those of CPI-613 (200 µM); 50 µM **TTCI** NPs showed an increase in apoptosis of up to 97.3% in BxPC3 cells. Therefore, this mitochondria-targeted prodrug nanoparticle platform provides a potential strategy for developing safe, targeting and efficient drug delivery systems for pancreatic cancer therapy.

## 1. Introduction

Pancreatic cancer, known as the “king of cancers”, is the fourth leading cause of cancer mortality in the world and its prognosis is cruel: the five-year survival rate remains lower than 10% [1,2]. CPI-613, a member of a novel class of anti-cancer lipoate derivatives, is an anti-mitochondrial metabolism agent which strongly induces apoptosis by disrupting mitochondrial metabolism, such as by changing mitochondrial enzyme activities and redox status in several types of cancer cells, especially in pancreatic cancer and small lung cancer cells [3,4]. CPI-613 has exhibited prominent antitumor activity against human pancreatic cancer in xenograft models, with low side-effects thanks to its specificity and selectivity for tumor cells [5,6,7]. CPI-613 has been approved as an orphan drug for the treatment of pancreatic cancer as well as other diseases by the U.S. Food and Drug Administration (FDA) [8].

Targeted drug delivery systems (DDSs) using passive targeting via the “enhanced permeability and retention (EPR) effect” or via active targeting for chemotherapy provide a beneficial strategy to improve antitumor efficiency and reduce side effects by promoting greater accumulation of delivered cargos at target sites [9]. Most DDS to date concentrate more on cellular internalization using receptors on the plasma membrane of certain cells which they specifically recognize and interact with [10]. However, the action sites of most clinically approved chemotherapeutic drugs (CPI-613, for instance) are focused on certain organelles within specific cells [11]. Herein, the DDS concentrates on certain organelles such as the nucleus and the mitochondria within specific cells, a method which has gradually garnered increasing interest for its further improvement the therapeutic effect [12].

It is well known that mitochondria are the powerhouse of most eukaryotic cells and play a crucial role in cellular metabolism. Mitochondria are responsible for various significant features, such as providing cellular energy by generating adenosine triphosphate (ATP), generation of reactive oxygen species (ROS), and regulating apoptotic cell death [13,14]. Mitochondria have been treated as interesting targets in organelle-targeted therapy, since they play pivotal roles in regulating cell survival and death. Thus, targeting mitochondria with therapeutic drugs presents plenty of advantages. Primarily, potential off-target toxic side-effects on normal tissues can be prevented if the drugs are targeted for delivery to the organelles. Furthermore, lower doses of drug administration could be approved by improving the bioavailability of the drugs at the target side, leading to maximized therapeutic efficiency [15,16]. Therefore, an increasing number of mitochondria-targeting DDS have been developed to promote active targeting via nanoplatform [15,17,18,19]. Among these, triphenylphosphonium (TPP), a lipophilic cation which has revealed a high affinity for mitochondria with about 1000-fold accumulation in this organelle [20], is one of the most widely used mitochondria targeting groups. The conjugation of TPP with therapeutic drugs has been developed to confirm mitochondria targeting [21,22,23]. However, until now few studies have been conducted on the targeted delivery of chemotherapeutic drugs such as CPI-613, whose action sites are focused on mitochondria.

Drug release behavior has an important impact on therapeutic efficiency and is one of the most vital factors to be considered in the design of a DDS. As mentioned above, mitochondria are the main centre for the production of ROS (for example, OH−), O_2_−, H_2_O_2_) as byproducts of ATP generation [24,25,26]. Therefore, the high levels of intracellular ROS in cancer cells could be faultlessly utilized as a unique cancer-related stimulus to mediate drug delivery. It has been reported that thioketal groups can be readily cleaved in ROS-abundant conditions [27,28,29], which has inspired us to develop ROS-responsive controlled drug release in mitochondria.

Considering the unique features of anti-mitochondrial metabolism agent (CPI-613) and the high levels of ROS in mitochondria, mitochondrial targeting ligand (TPP) and biodegradable thioketal linkages, it is desirable to conjugate CPI-613 and TPP with biodegradable ROS-responsive thioketal linkages in order to exploit novel mitochondrial targeting DDS. In this study, we present a smart nanoplatform for active mitochondrial targeting and ROS-responsive drug release. First, the mitochondrial targeting ligand TPP was conjugated onto thioketal linkages (TPP-TK). Then, CPI-613 was combined by esterification (TTP-TK-CPI-613). Aiming for better tumor targeting and good biocompatibility, DSPE-PEG2000 was employed as an assistant component in order to increase the accumulation in the tumor via the EPR effect. This novel nanoplatform shows the following unique properties: (1) The hydrophilic PEG2000 shell can stabilize the DDS, prolong circulation in blood, and increase accumulation in the tumor; (2) TPP can facilitate the targeted delivery of CPI-613 to its acting site; (3) The TK-containing linker can promote ROS-triggered drug release; and finally (4) It has good biocompatibility and is highly biodegradable. Therefore, the smart nanoplatform will be able to encapsulate anti-mitochondrial metabolism agent (CPI-613), contain a TPP passive mitochondrial targeting moiety, and release ROS-triggered drugs to improve drug delivery ability and therapeutic efficacy in pancreatic cancer cell lines. Overall, the prepared multifunctional DDS exhibits multiple advantages including an efficient TPP-mediated mitochondria-targeted delivery method, good biocompatibility and biodegradability, a rapid ROS-triggered drug release, and great potential for chemotherapy in pancreatic cancer.

## 2. Materials and Methods

All chemicals and reagents were obtained commercially and were used as received. Mercaptoacetic acid was purchased from Aladdin Reagent Company (Shanghai, China). Cholesterol and rhodamine B base were purchased from BBI Life Sciences (Shanghai, China). CPI-613 was purchased from Bidepharm Reagent Company (Shanghai, China). 1,2-distearoyl-sn-glycero-3-phosphoethanolamine-N-[methoxy (polyethylene glycol)-2000] (ammonium salt) (DSPE-PEG2000) was purchased from Avanti Polar Lipids (Birmingham, AL, USA). The products were purified by Biotage Isolera™ Spektra Systems. A Bruker 400 spectrometer was used for recording ^1^H and ^13^C NMR. HPLC-MS analyses were performed on a LCMS-2020 instrument (Shimadzu, Kyoto, Japan) using the following conditions: Shim-pack VPODS C_18_ column (reverse phase, 150 mm × 2.0 mm); 80% acetonitrile and 20% water over 6.0 min; flow rate of 0.4 mL/min; UV photodiode array detection from 200 to 300 nm.

3-(4,5-Dimethylthiazol-2-yl)-2,5-diphenyltetrazolium bromide (MTT) was purchased form Sangon Biotech (A600799-0005, Shanghai, China). Mito-Tracker Green and Annexin V-FITC Apoptosis Detection Kit were purchased from Beyotime Biotechnology (Shanghai, China).

### 2.1. Synthesis of Prodrug ***TTCI***

#### 2.1.1. Synthesis of Compounds **1** and **2**

Compounds **1** and **2** were obtained according to the previous literature [30,31]. Briefly, 3-mercptopropionic acid (5 g, 47.2 mmol), anhydrous acetone (1.3 g, 21.46 mmol), and trifluoroacetic acid (20 μL) were mixed in a 100 mL round-bottom flask. After stirring overnight at room temperature, a white solid was precipitated. The solid was filtered and washed several times with ice-cold deionized water and hexane. After drying in vacuum, compound 1 was obtained as a white crystalline powder (yield: 90%). 1H NMR (CDCl_3_, 400 MHz): δ (ppm) = 2.92–2.88 (t, 4H, –CH_2_–), 2.70–2.66 (t, 4H, –CH_2_–), 1.60 (s, 6H, –CH_3_); 13C NMR (CDCl_3_, 100 MHz): δ (ppm) = 178.44, 56.50, 33.50, 30.73, 24.86. 

Compound **2** was obtained by the reduction of LiAlH4 on compound **1**. Briefly, compound **1** (1.0 g, 4 mmol) was dissolved in 30 mL of anhydrous THF under magnetic stirring. LiAlH4 (0.9 g, 24 mmol) was slowly added into the reaction mixture. The mixed reaction solution was heated to reflux for 2 h. Then, 0.9 mL NaOH (*w*/*w* 15%) aqueous solution and 0.9 mL water were added to the flask. After filtration, the solvent was concentrated and dried in vacuum overnight to give compound **2** as a colorless oil (yield: 87%). 1H NMR (CDCl_3_, 400 MHz): δ (ppm) = 3.77–3.74 (t, 4H, –CH_2_–), 2.77–2.74 (t, 4H, –CH_2_–), 1.89–1.82 (m, 4H, –CH_2_–), 1.61 (s, 6H, –CH_3_). ^13^C NMR (CDCl_3_, 100 MHz): δ (ppm) = 172.93, 135.28, 133.46, 133.37, 130.67, 130.55, 118.17, 117.31,63.70, 61.72, 56.01, 38.6, 31.94, 30.90, 28.21, 26.70, 26.56, 17.85.

#### 2.1.2. Synthesis of Compound **3**

3-Carboxypropyltriphenylphosphonium bromide (637 mg, 1.483 mmol) was dissolved in DCM; 4-Dimethylamino-pyridine (DMAP, 217 mg, 1.78 mmol) and *N*,*N*-dicyclohexyl-carbodiimide (DCC, 367 mg, 1.78 mmol) were added successively at 0 °C for 30 min. Then, compound **2** (400 mg, 1.78 mmol) was added to the mixed solution. After reaction at room temperature overnight, the reaction solution was cooled to −20 °C. Filtration was performed and the filtrate was concentrated. After purification by column chromatography over silica gel eluting with a gradient of CH_3_OH/CH_2_Cl_2_ (0 to 5%), compound **3** was obtained as colorless oil (yield: 84%). 1H NMR (CDCl_3_, 400 MHz): δ (ppm) = 7.90–7.64 (m, 15H, –CH–), 4.21–4.11 (q, 2H, –CH_2_–), 3.36–3.22 (m, 2H, –CH_2_–), 2.73–2.59 (m, 6H, –CH_2_–), 1.94–1.84 (m, 6H, –CH_2_–), 1.84–1.75 (m, 2H, –CH_2_–), 1.57 (s, 6H, –CH_3_).

#### 2.1.3. Synthesis of Prodrug **TTCI**

CPI-613 (50 mg, 0.128 mmol) was dissolved in anhydrous DCM; 4-Dimethylamino-pyridine (DMAP, 19 mg, 0.155 mmol) and N, N-dicyclohexyl- carbodiimide (DCC, 32 mg, 0.155 mmol) were added successively at 0 °C for 30 min. Then, compound **3** (98 mg, 0.155 mmol) was added to the mixed solution. After stirring at room temperature overnight, the reaction solution was cooled to -20 °C. Filtration was performed and the filtrate was concentrated. After purification by column chromatography over silica gel eluting with a gradient of CH_3_OH/CH_2_Cl_2_ (0 to 7%), prodrug **TTCI** was obtained as colorless oil (yield: 41%). 

1H NMR (CDCl_3_, 400 MHz): δ (ppm) = 7.90–7.69 (m, 15H, –CH–), 7.31–7.27 (m, 5H, –CH–), 7.25–7.19 (m, 2H, –CH–), 4.13–4.12 (q, 4H, –CH_2_–), 3.66–3.64 (d, 4H, –CH_2_–), 2.89 (t, 2H, –CH_2_–) 2.66–2.62 (t, 4H, –CH_2_–), 2.60–2.53 (t, 1H, –CH_2_–), 2.53–2.46 (t, 2H, –CH_2_–), 2.24–2.23 (t, 2H, –CH_2_–), 1.92–1.88 (m, 6H, –CH–), 1.89–1.82 (m, 4H, –CH_2_–), 1.73–1.71 (m, 2H, –CH_2_–), 1.58 (s, 6H, –CH_3_), 1.53–1.49 (t, 2H, –CH_2_–), 1.47–1.41 (t, 2H, –CH_2_–). ^13^C NMR (CDCl_3_, 100 MHz): δ (ppm) = 173.52, 173.24, 138.65, 138.49, 134.97, 133.83, 133.73, 130.50, 130.38, 128.89, 128.85, 128.50, 128.45, 126.97, 126.93, 118.74, 117.89, 63.64, 63.23, 56.01, 44.28, 36.38, 35.13, 34.45, 34.12, 30.94, 28.73, 28.65, 26.61, 26.16, 24.74, 14.13. HRMS (ESI): *m*/*z* calculated for C_53_H_66_O_4_PS_4_+ [M] 925.3576; found 925.3582. 

#### 2.1.4. Synthesis of Cholesterol Analogue **TTCh**

Cholesterol (1.0 g, 2.59 mmol) and succinic anhydride (0.776 g, 7.76 mmol) were dissolved in DCM at 0 °C; 1,8-Diazabicyclo (5.4.0) undec-7-ene (DBU, 1.179 g, 7.76 mmol) was slowly added to the reaction solution, and the reaction was stirred at room temperature for 4 h. The solution was acidified with 1% aqueous HCl solution, and DCM was added. The organic layer was collected and evaporated. The product was obtained after purification by column chromatography over silica gel eluting with a gradient of CH_3_OH/CH_2_Cl_2_ (0 to 6%). The upper product (75 mg, 0.155 mmol) was dissolved in anhydrous DCM; 4-Dimethylamino-pyridine (DMAP, 19mg, 0.155 mmol) and *N,N*-dicyclohexyl-carbodiimide (DCC, 32 mg, 0.155 mmol) were added successively at 0 °C for 30 min. Then, compound 3 (98 mg, 0.155 mmol) was added to the mixed solution. After stirring at room temperature overnight, the reaction solution was cooled to −20 °C. Filtration was performed and the filtrate was concentrated. After purification by column chromatography over silica gel eluting with a gradient of CH_3_OH/CH_2_Cl_2_ (0 to 6%), **TTCh** was obtained as colorless oil (yield: 62%). 

1H NMR (CDCl_3_, 400 MHz): δ (ppm) = 7.85–7.65 (m, 15H, -CH-), 5.40–5.32 (m, 1H, –CH–), 4.66–4.55 (m, 1H, –CH–), 4.24–4.11 (t, 4H, –CH_2_–), 3.40–3.28 (d, 2H, –CH_2_–), 2.75–2.54 (m, 10H, –CH_2_–), 2.35–2.26 (d, 2H, –CH_2_–), 2.06–1.79 (m, 12H), 1.58 (s, 6H, –CH_3_), 1.60–0.76 (m, 35H). 13C NMR (CDCl_3_, 100 MHz): δ (ppm) = 172.89, 172.33, 171.70, 139.60, 135.29, 133.48, 133.38, 130.67, 130.55, 122.68, 118.20, 117.35, 74.36, 63.79, 63.61, 56.70, 56.14, 55.98, 50.03, 42.31, 39.73, 39.52, 38.06, 36.96, 36.59, 36.19, 35.79, 32.73, 31.91, 31.85, 30.90, 29.70, 29.47, 29.19, 28.54, 28.41, 28.23, 28.01, 27.74, 26.56, 24.29, 23.89, 22.82, 22.57, 21.45, 21.03, 19.32, 18.72, 17.89, 11.86. HRMS (ESI): *m*/*z* calculated for C_62_H_88_O_6_PS_2_+ [M] 1023.5754; found 1023.5779.

### 2.2. Preparation of Nanoparticles (NPs)

**TTCI**, **TTCI**/**Rho** and **TTCh** NPs were formed using the filming–rehydration method. The additional lipid 1,2-distearoyl-sn-glycero-3-phosphoethanolamine-N-[methoxy (polyethylene glycol)-2000] (ammonium salt) (DSPE-PEG2000) was used to form nanoparticles. First, **TTCI** NPs with the different proportions were made up by prodrug **TTCI** and DSPE-PEG2000 in 1:x (mol:mol), and the x was range 0.05–0.30. The prodrugs **TTCI** and DSPE-PEG2000 were mixed in a little glass vial and dissolved in 2.5 mL anhydrous chloroform/methanol solution (3/1, *v*/*v*). The thin films were obtained by slowly rotary-evaporating the solvent and further dried under vacuum overnight. Then, thin films were hydrated with 2.5 mL of PBS buffer (10 mM, pH 7.4) at 70 °C for 30 min. After 10 min tip-sonication (3 s on, 3 s off), **TTCI** NPs were obtained. The same method was used to prepare **TTCh** NPs, in which **TTCh** and DSPE-PEG2000 were only in 1:0.2 (mol:mol). In order to observe intracellular distribution, **TTCI**/**Rho** NPs were also prepared using the same method. **TTCI**/**Rho** NPs were made up with **TTCI**, DSPE-PEG2000 and Rhodamine B base with 1:0.2:0.2 (mol:mol:mol). The final concentration of prodrug **TTCI** or **TTCh** was 1 mM. If not specified, **TTCI** NPs were made up by **TTCI** and DSPE-PEG2000 with 1:0.2 (mol:mol).

### 2.3. Characterization of ***TTCI*** NPs

A dynamic light scattering (DLS) Zetasizer nano zsp instrument (Malvern instruments Led) was used to measure the diameter distribution at room temperature. The size of **TTCI** NPs with different proportions was first characterized. 200 µL **TTCI** NPs (different proportions, 1mM) were diluted with deionized water to produce 1 mL aqueous solution. The diameter distribution of the diluted aqueous solution was measured at room temperature.

The morphology of **TTCI** NPs (**TTCI**:DSPE-PEG2000 = 1:0.2 (mol:mol)) was observed by transmission electron microscopy (TEM) on a JEM-1400plus system (JEOL, Japan); 30 µL **TTCI** NPs (1 mM) was diluted to 100 µL aqueous solution and mixed together. The diluted solution was applied to a copper grid and 0.2% (*w*/*v*) phosphotungstic acid aqueous solution was used to stain the samples.

### 2.4. ROS-Triggered Disassembly Studies of ***TTCI*** NPs

The 1 mL mixed aqueous solutions containing 200 µL **TTCI** NPs under H_2_O_2_ (200 mM or 700 mM) were incubated at 37 °C. At appropriate intervals, the size changes were monitored by DLS.

### 2.5. Cell Culture

Human pancreatic cancer cell lines AsPC-1, PANC-1 and BxPC3 were purchased from the American Type Culture Collection (ATCC, Rockville, MD, USA). AsPC-1 and PANC-1 cells were cultured in RPMI1640 medium containing 10% fetal bovine serum (FBS). The BxPC3 cells were cultured in DMEM medium containing 10% FBS. Cells were cultivated at 37 °C in a humidified incubator supplied with 5% CO_2_.

### 2.6. Cell Viability Assay

The effect on pancreatic cancer cells of **TTCI** NPs in different proportions was measured with an MTT assay. AsPC-1 and PANC-1 cells were counted and seeded into 96-well plates (6000 cells/well) with 100 µL cell medium and incubated for 24 h. The cells were treated with another 100 μL of complete medium containing increasing concentrations of **TTCI** NPs in different proportions. After incubation for 48 h, 20 μL MTT solution (5 mg/mL) was added. After 4 h incubation, the original medium was removed and 200 μL dimethylsulfoxide (DMSO) was added to each well. The plate was shaken on the table shaker for 10 min. Next, the absorbance was measured at 570 nm by a microplate reader (Bio-Tek, Winooski, VT, USA). All experiments were repeated three times.

The cell viability of **TTCI** NPs (**TTCI**:DSPE-PEG2000 = 1:0.2 (mol:mol)) and **TTCh** NPs (**TTCh**:DSPE-PEG2000 = 1:0.2 (mol:mol)) on AsPC-1 and PANC-1 cells was determined by the same method. The cells were treated with complete medium containing increasing concentrations of **TTCI** NPs or **TTCh** NPs.

The cell viability of **TTCI** NPs (**TTCI**:DSPE-PEG2000 = 1:0.2 (mol:mol)) and CPI-613 on AsPC-1, PANC-1 and BxPC3 was also determined by the same method. The cells were incubated with complete medium containing increasing concentrations of **TTCI** NPs and CPI-613.

### 2.7. Cell Proliferation and Growth

The effect of **TTCI** NPs on proliferation and growth in pancreatic cancer cells at different time points was observed by using the High Content analysis system (PerkinElmer, Waltham, MA, USA). PANC-1 and BxPC3 cells were seeded in a CellCarrier 96-well microplates (PerkinElmer) overnight and treated with PBS, **TTCI** NPs or CPI-613. Then, the 96-well microplate was placed into the High Content analysis system for incubation. At appropriate intervals, digital phase contrast images of cells were captured. The scale bar was 100 µm.

### 2.8. Intracellular Distributions

BxPC3 cells were seeded in CellCarrier 96-well microplates overnight and treated with **TTCI**/**Rho** NPs (20 µM). Intracellular distributions of **TTCI**/**Rho** NPs for different time (0.5, 2 and 4 h) were observed by using the High Content analysis system. Average fluorescence intensity of the picture was quantified by Image J software (Image J 1.49, Wayne Rasband National Institutes of Health, Bethesda, MD, USA).

### 2.9. Mitochondria Targeted Imaging

BxPC3 cells were seeded in CellCarrier 96-well microplates overnight and treated with **TTCI**/**Rho** NPs (20 µM) for 0.5 or 4 h. The cells were stained with Mito-Tracker Green (λex = 488 nm, λem = 516 nm) for 30 min. Images were acquired using the High Content analysis system. The colocalization in the magnification box was quantified by Image J software.

### 2.10. Cell Apoptosis Assay 

Apoptosis induced by the **TTCI** NPs in BxPC3 cells was analyzed by flow cytometry (Becton Dickinson, AccuriTM C6, Franklin Lakes, NJ, USA). PBS and CPI-613 were used as a control. The Annexin V-FITC Apoptosis Detection Kit was used according to the manufacturer’s instructions. Briefly, the BxPC3 cells were treated with PBS, **TTCI** NPs or CPI-613 for 48 h. The cells were harvested and washed twice with cold PBS and then stained with PI and annexin V-FITC for 15 min at room temperature. The PI/annexin V-FITC signal was determined using flow cytometry.

## 3. Results and Discussion

### 3.1. Synthesis and Characterization of the Lipid Prodrug

**Scheme 1:**
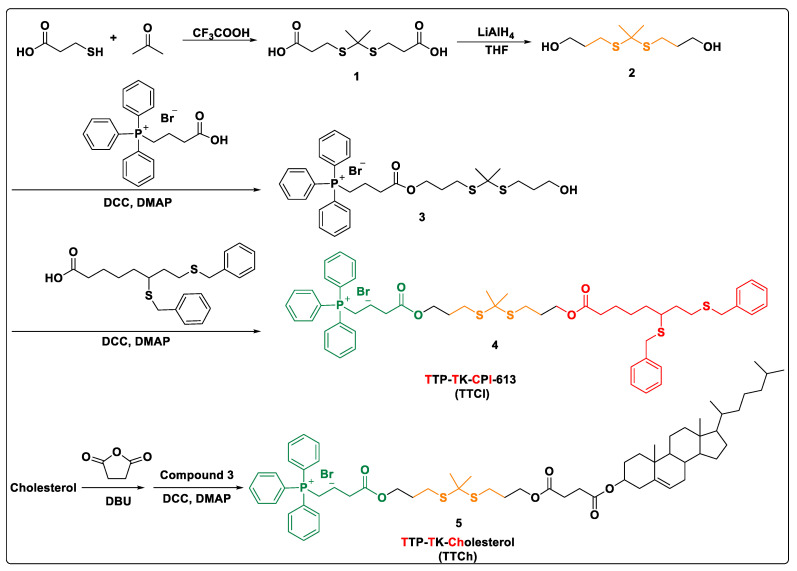
Synthetic routes of target lipid prodrugs and analogues.

The lipid prodrug was synthesized by conjugating model drug CPI-613 and mitochondrial targeting ligand TPP with thioketal skeleton, as shown in Scheme 1. In brief, thioketal linkage (compound **2**) was prepared by reduction of compound **1**, which was synthesized by 3-mercaptopropionic acid and anhydrous acetone under a catalytic amount of trifluoroacetic acid. Then the (3-carboxypropyl) triphenyl phosphonium bromide (targeting ligand TPP) was conjugated onto one end of skeleton **2** in the presence of DCC and DMAP to give precursor **3**. Subsequently, the target mitochondria-targeted prodrug **4** (represented as **TTCI** based on the abbreviation of different functional groups) was obtained by coupling anticancer drug molecule CPI-613 to the other end of the linkage under the same conditions. For comparison, cholesterol analogue **TTCh** using cholesterol instead of CPI-613 was also prepared through a similar method. All novel compounds in each step were purified and their structures were confirmed by ^1^H NMR, ^13^C NMR and HRMS.

### 3.2. Preparation and Characterization of CPI-613-Prodrug NPs

**Figure 1:**
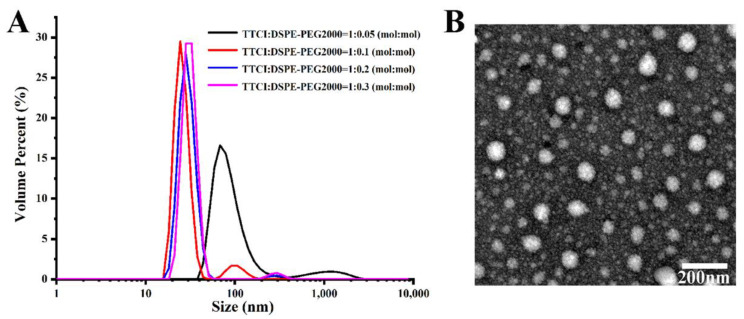
(**A**) Size distribution of CPI-613-prodrug NPs constructed with different molar ratios of **TTCI**:DSPE-PEG2000. (**B**) The TEM image of CPI-613-prodrug NPs constructed with **TTCI**:DSPE-PEG2000 at a molar ratio of 1:0.2. Scale bar = 200 nm.

After the successful synthesis of **TTCI** and **TTCh**, the self-assembly abilities of **TTCI** were evaluated. Polyethylene glycol (PEG) in particular has been widely used to modify the surface of nanoparticles because it has several unique advantages such as hydrophilicity, non-antigenicity and non-immunogenicity. Hence, nanoparticles can avoid uptake by macrophages and plasma opsonization when their surface is engineered using PEG. This results in the realization of prolonged circulation time, improved nanoparticles and increased tumor targeting via the “enhanced permeability and retention” (EPR) effect [32,33]. Therefore, DSPE-PEG2000, which has been approved for clinical use by the U.S. Food and Drug Administration (FDA), was added to improve the performance of liposome NPs self-assembled via **TTCI**. CPI-613-prodrug NPs were constructed with **TTCI**:DSPE-PEG2000 at different molar ratios using the thin film hydration method [34]. First, the particle sizes of the prepared NPs with different composition proportions were measured by dynamic light scattering (DLS). As shown in Figure 1A, prodrug NPs with a lower content of DSPE-PEG2000 (**TTCI**:DSPE-PEG2000 = 1:0.05) had a larger particle size (~70 nm). With increased of DSPE-PEG2000 content, prodrug NPs which were constructed with **TTCI**:DSPE-PEG2000 at molar ratios of 1:0.1, 1:0.2 and 1:0.3 had much smaller particle sizes (~30 nm), with no obvious difference between them. The PDI values of these NPs were 0.256, 0.407, 0.468 and 0.669, respectively. The PDI values increased with the increase of DSPE-PEG2000. Subsequently, MTT-based cell viability assays were carried out in PNAC-1 and AsPC-1 cells in order to investigate the effect of composition ratio on cytotoxicity. It was found that prodrug NPs constructed with **TTCI**:DSPE-PEG2000 at a molar ratio of 1:0.2 exhibited the best in vitro antitumor activity when the concentration of CPI-613 was over 10 µM, in both cell lines (Figure S1). Therefore, CPI-613-prodrug NPs which were constructed with **TTCI**:DSPE-PEG2000 at a molar ratio of 1:0.2 were chosen for the following model example. Herein, transmission electron microscopy (TEM) was used to visually study the morphological characteristics of the prodrug NPs constructed with **TTCI**:DSPE-PEG2000 at a molar ratio of 1:0.2. The image in Figure 1B shows that those NPs have spherical distribution, with diameters ranging from 20 nm to 50 nm. What is more, **TTCI** NPs reveal good stability (Figure S2) and have a 0.505 mV surface charge (Figure S3).

### 3.3. Measurement of ROS-Responsive ***TTCI*** NPs Degradation In Vitro

**Figure 2:**
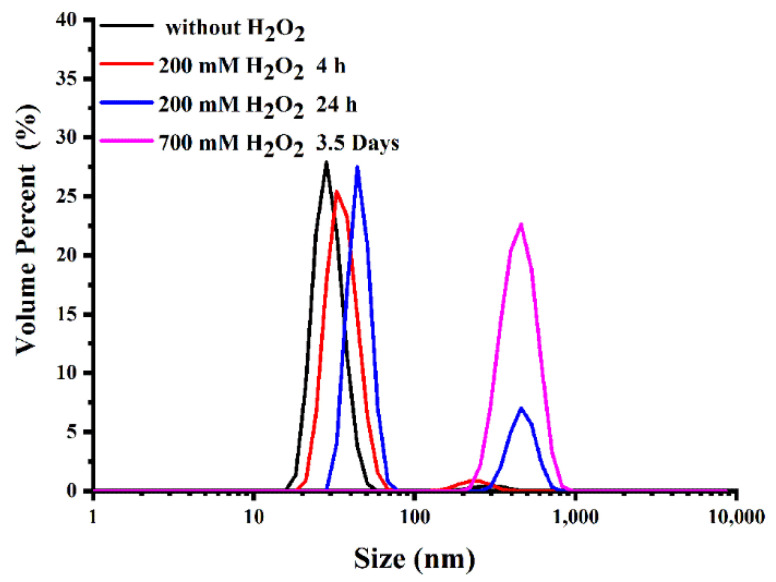
Size distribution of **TTCI** NPs after treatment with various H_2_O_2_ concentrations for different times.

The thioketal linkers between TPP and CPI-613 can respond to ROS. To investigate this responsive behavior, the size changes of **TTCI** NPs were monitored over time after treatment with different concentrations of ROS. As shown in Figure 2, at a low ROS concentration (200 mM H_2_O_2_ in the presence of Fe^2+^) for 4 h incubation, the size changes of **TTCI** NPs were not obvious. However, with the extension of incubation time to 24 h, the size of the **TTCI** NPs increased and a new peak appeared at about 400 nm, indicating the destruction of NPs. The size of **TTCI** NPs increased from 30 nm to about 400 nm after incubation with 700 mM H_2_O_2_ for 3.5 days, which can be attributed to the degradation of the thioketal group triggered by ROS, resulting in the separation of TPP and CPI-613 from the **TTCI**. Similar results were found with **TTCh** NPs (Figure S4). This feature may enable more CPI-613 molecules to be released at the active site, further improving therapeutic efficacy.

### 3.4. Cellular Internalization and the Specific Mitochondria-Targeting Function of the ***TTCI*** NPs

For evaluation of the cellular uptake behavior of **TTCI** NPs, Rhodamine B base (or Nile Red), a fluorescent dye, was encapsulated in **TTCI** NPs to form Rhodamine B-loaded **TTCI** NPs (**TTCI**/**Rho** NPs) to permit indirect observation with a fluorescence microscope, as the prodrug **TTCI** has no inherent fluorescence. Confocal laser scanning microscopy (CLSM) with a high content analysis system was applied in order to visually examine the internalization and intracellular location of **TTCI**/**Rho** NPs at the concentration of 20 µM in Pancreatic Carcinoma line-3 (BxPC3 cells). The fluorescence intensity detected in cells can clearly indicate the concentration of these systems internalized into the cells. As shown in Figure S5A, a considerable amount of the yellow fluorescent signal of Rhodamine B could be observed after 30 min, indicating rapid and high cellular uptake of **TTCI**/**Rho** NPs. It can be also seen that the fluorescence signals in the cells increased gradually with the extension of incubation time (from 0.5 h to 4 h). The mean intensity of Rhodamine B fluorescence in the images at different incubation times was calculated using imageJ software (Figure S5B). The mean fluorescence intensity increased gradually with the extension of incubation time, which was mostly consistent with the visual results. This suggests that **TTCI**/**Rho** NPs exhibited fast and efficient cellular internalization, which is necessary for the following delivery process.

**Figure 3:**
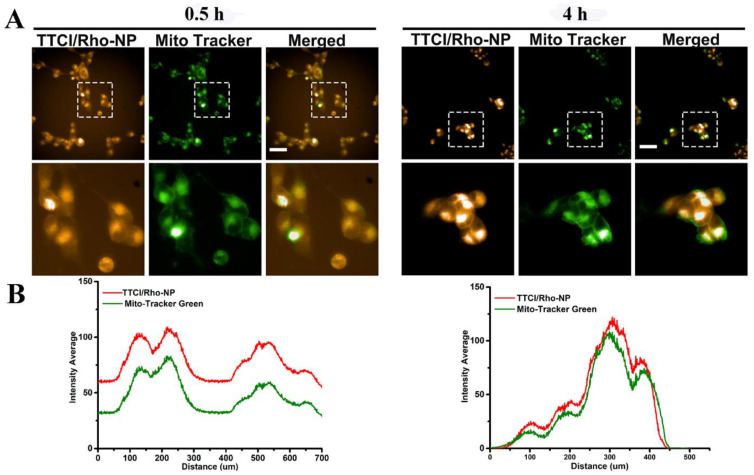
Mitochondria-targeting ability of **TTCI**/**Rho** NPs in vitro: Images of the BxPC3 cells treated with **TTCI/Rho** NPs at a concentration of 20 µM for 0.5 and 4 h, captured by high content analysis system-operetta CLS^TM^. For each row, from left to right: **TTCI/Rho** NPs (546 nm excitations); mitochondria stained by MitoTracker Green (488 nm excitation); merged image (**A**). Scale bars: 50 µm. The cross-section fluorescence intensity of Rhodamine B and MitoTracker Green in the enlarged regions analyzed using imageJ software (**B**).

Subsequently, the possibility of targeting mitochondria was further investigated using mitochondria and **TTCI** NPs labeled/stained with MitoTracker Green and Rhodamine B or Nile Red (NR), respectively. BxPC3 cells were incubated with **TTCI**/**Rho** NPs at a concentration of 20 µM for 0.5 h and 4 h and with the commercial mitochondrial dye MitoTracker Green at 37 °C for 0.5 h, respectively. The images were captured by high content analysis system-Operetta CLS^TM^ after 0.5 and 4 h of incubation (Figure 3A). It was found that after 0.5 and 4 h the cells treated with **TTCI**/**Rho** NPs showed obvious co-localization between the mitochondrial probes MitoTracker Green and Rhodamine B encapsulated by **TTCI** NPs. Abundant colocalization fluorescent signals were especially found after 4 h incubation, indicating that the targeted prodrugs can easily realize our purpose for preferably targeting internalized mitochondria in living cells. The cross-section fluorescence intensity of Rhodamine B and MitoTracker Green in the enlarged regions was further analyzed using imageJ software (Figure 3B). It was found that the fluorescence change trend of two dyes was the same, further confirming the excellent co-localization of mitochondria and delivered cargos. Similar results were obtained for **TTCI**/**NR** NPs (Figure S6). These observations reveal the potential of TPP modified prodrugs such as **TTCI** to deliver CPI-613 to mitochondria, and to further exert therapeutic effects after accumulating in mitochondria.

### 3.5. In Vitro Antitumor Activity

It has been reported that CPI-613 exhibits prominent antitumor activity, especially against human pancreatic cancer [1,3,4]. Therefore, in this work, three human pancreatic cell lines (PANC-1, AsPC-1 and BxPC3) were used as the models for the MTT assay. Firstly, the cytotoxicity of nanocarriers without encapsulation of CPI-613 was measured to prove that the drug carriers are biocompatible. A lipid prodrug counterpart was synthesized by simply replacing the CPI-613 molecule with biocompatible cholesterol, represented as **TTCh** (TPP-TK-Cholesterol) for short. The **TTCh** NPs were constructed using the same method as **TTCI** NPs, with **TTCh**:DSPE-PEG2000 at a molar ratio of 1:0.2. The cytotoxicity of **TTCI** NPs and **TTCh** NPs against AsPC-1 and PANC-1 cells were investigated at various concentrations. As shown in Figure S7, the **TTCh** NPs without loaded CPI-613 presented little cytotoxicity in both AsPC-1 and PANC-1 cells, and cellular viability remained over 90% for concentrations in the range of 0.1 µM to 50 µM. In contrast, **TTCI** NPs containing CPI-613 showed significant cytotoxicity in both cells when the concentration was greater than 20 µM. The cell viability percentages remained at about 20% when treated with 50 µM **TTCI** NPs. The above results indicate that the CPI-613 conjugated to the prodrug plays a vital role in cytotoxicity.

**Figure 4:**
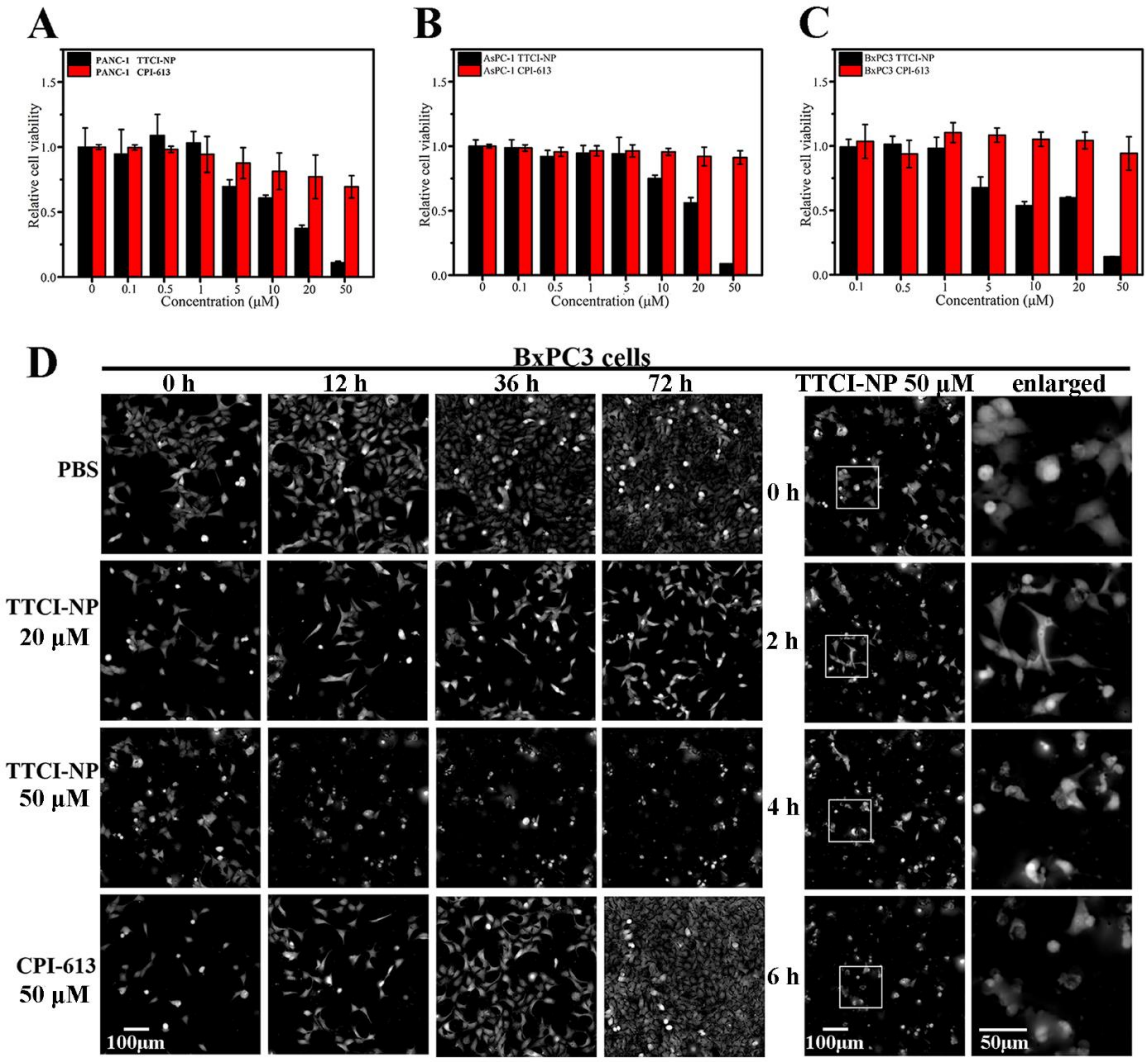
In vitro cytotoxicity of free CPI-613 and CPI-613-loaded **TTCI** NPs at various concentrations against PANC-1 cells (**A**), AsPC-1 cells (**B**), and BxPC3 cells (**C**) after 48 h incubation. Data represent mean ± SD (*n* = 3). Digital phase contrast images of living BxPC3 cells (**D**) treated with PBS, 20 µM **TTCI** NPs, 50 µM **TTCI** NPs and 50 µM CPI-613 at different times. Digital phase contrast images were captured by high content analysis system-operetta CLS^TM^. Scale bar: 100 µm and 50 µm (for enlarged images).

Subsequently, these three cell lines were used to investigate the in vitro anticancer efficiency of CPI-613-loaded supramolecular nanoplatforms. As shown in Figure 4A–C, after three kinds of human pancreatic cells were co-incubated with **TTCI** NPs at different CPI-613 concentrations, **TTCI** NPs presented dose-dependent cytotoxicity against PANC-1, AsPC-1 and BxPC3 cells, and the cell viabilities decreased obviously with the concentration of CPI-613, increasing from 10 µM to 50 µM. In contrast, the cellular viability remained over 90% for pure CPI-613 in all cell lines even with a concentration of drug up to 50 µM, indicating that CPI-613 encapsulated with **TTCI** NPs had a more significant ability to kill pancreatic cancer cells than free CPI-613. Meanwhile, the IC_50_ values of **TTCI** NPs in the three cells were around 20–30 µM, which was far lower than that of CPI-613 (reported around 200 µM [1]). Such a dramatic difference in IC_50_ values reveals the successful targeting effect and ROS-responsive release of **TTCI** NPs, which facilitated the accumulation of CPI-613 at its acting site mitochondria. Moreover, the above results can also be supported by the morphology and number of living cells for PBS: 20 µM of **TTCI** NPs, 50 µM of **TTCI** NPs and 50 µM of free CPI-613, respectively (Figure 4D). The high content analysis system-operetta CLS^TM^ was used to assess BxPC3 cell proliferation. The results showed that it induced a dramatic decrease in the total number of cells with 50 µM of **TTCI** NPs. However, cell proliferation was not affected by PBS and 50 µM of free CPI-613. Atrophy and deformation of PANC-1 cells appeared after 2 h co-incubation with 50 µM of **TTCI** NPs. Similar results were found in PANC-1 cells (Figure S8). The IC_50_ values of **TTCI** prodrug and **TTCI** NPs were not significantly different, and digital phase contrast images of living BxPC3 cells showed that it induced a dramatic decrease in the total number of cells with both 50 µM of **TTCI** prodrugs and **TTCI** NPs (Figure S9). However, the **TTCI** prodrugs are practically insoluble (dissolved in DMSO for biological experiments), which will severely restrict biological application. The evidence intuitively revealed that **TTCI** NPs had much better in vitro anticancer capacity. Therefore, all the above results imply that encapsulated CPI-613 **TTCI** NPs can be efficiently delivered to mitochondria and released in the ROS-enriched environment in mitochondria, resulting in an accumulation of therapeutic molecules which can efficiently inhibit the proliferation of pancreatic cancer cells.

### 3.6. Cell Apoptosis

**Figure 5:**
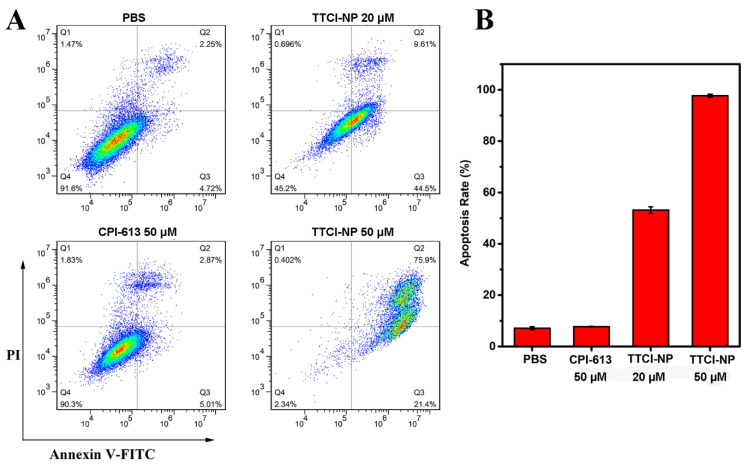
The effects on apoptosis of free CPI-613 and CPI-613-loaded **TTCI** NPs at various concentrations. BxPC3 cells were treated with 50 µM CPI-613, 20 µM **TTCI** NPs and 50 µM **TTCI** NPs for 48 h and apoptosis was determined by flow cytometry with Annexin V-FITC/PI staining (**A**). The apoptosis rate (**B**) following treatment with various therapeutics.

CPI-613 could induce pancreatic cancer apoptosis by disrupting mitochondrial metabolism [4]. Therefore, low cytometry analysis was performed after FITC Annexin V/PI staining in order to further assess the apoptosis of BxPC3 cells induced by various therapeutics. As such, BxPC3 cells were treated with PBS, 50 µM CPI-613, 20 µM **TTCI** NPs and 50 µM **TTCI** NPs as described above. After that, cell apoptosis was detected after further incubation for 48 h. The results showed that the ratio of apoptosis to cells was 7.88%, 54.11% and 97.3% as induced by 50 µM CPI-613, 20 µM **TTCI** NPs and 50 µM **TTCI** NPs, respectively (Figure 5). These data distinctly indicate that **TTCI** NPs can induce apoptosis in pancreatic cancer cells to a dramatic degree, especially at a concentration of 50 µM. This observation of in vitro antitumor activity and cell apoptosis together shows the huge potential of **TTCI** nanoplatforms for pancreatic cancer treatment.

## 4. Conclusions

In summary, a mitochondria-targeted and ROS-responsive prodrug **TTCI** was developed by conjugating the model drug CPI-613 and mitochondrial targeting ligand TPP with a thioketal linker. The prodrugs can self-assemble into stable nanoparticles (**TTCI** NPs) together with DSPE-PEG2000, with resulting unique properties including effective mitochondrial targeting, ROS-cleaving capability and robust therapeutic performance. In addition, the smart nanoplatform showed enhanced therapeutic effects on pancreatic cancer, mainly due to higher accumulation of nanoparticles in mitochondria induced by mitochondrial targeting, facilitating the targeted delivery of CPI-613 to its active site. The results with respect to in vitro antitumor activity and cell apoptosis revealed that the IC_50_ values of **TTCI** NPs in three pancreatic cancer cell lines were around 20 µM–30 µM, which was far lower than that of CPI-613 (200 µM), and that 50 µM **TTCI** NPs could increase apoptosis by as much as 97.3% in BxPC3 cells. Therefore, our study provides a potential strategy for developing safe, targeted and efficient drug delivery systems for use in pancreatic cancer therapy.

## Supplementary Materials

The following are available online at https://www.mdpi.com/article/10.3390/nano11112875/s1. Figure S1: In vitro cytotoxicity of **TTCI** NPs constructed with different molar ratios of **TTCI**:DSPE-PEG2000 in PNAC-1 (A) and AsPC-1 (B) cells treated with various concentrations for 48 h. Data represent mean ± SD (*n* = 3). Figure S2: Mean particle sizes of **TTCI** NPs constructed with **TTCI**:DSPE-PEG2000 at a molar ratio of 1:0.2 at different time points (DLS at room temperature). Data represent mean ± SD (*n* = 3). Figure S3: Zeta-potentials of **TTCI** NPs constructed with **TTCI**:DSPE-PEG2000 at a molar ratio of 1:0.2 (DLS at room temperature). Figure S4: Size distribution of **TTCh** NPs after treatment with 700 mM H_2_O_2_ for different times. Figure S5: Images of the BxPC-3 cells treated with Rhodamine B-loaded **TTCI**/**Rho** NPs at a concentration of 20 µM for 0.5, 2, and 4 h, respectively. For each row, left: **TTCI**/**Rho** NPs; middle: bright field; right: merged image. The images were captured by high content analysis system-operetta CLS^TM^ (A). Scale bars: 50 µm. The mean intensity of rhodamine fluorescence in the images at different incubation times as calculated using imageJ software (B). Figure S6: Mitochondria-targeting ability of **TTCI**/**NR** NPs in vitro: Images of the BxPC3 cells treated with **TTCI**/**NR** NPs at the concentration of 20 µM for 0.5, 2 and 4 h captured by high content analysis system-operetta CLS^TM^. For each row, from left to right: **TTCI**/**NR** NPs; mitochondria stained by MitoTracker Green; merged image. Scale bars: 50 µm. Figure S7: Cell viability of AsPC-1 (A) and PANC-1 (B) cells after being treated with different concentrations of **TTCI** NPs and **TTCh** NPs for 48 h. Data represent mean ± SD (*n* = 3). Figure S8: Digital phase contrast images of living BxPC-3 cells (D) treated with PBS, 20 µM **TTCI** NPs, 50 µM **TTCI** NPs and 50 µM CPI-613 at different times. Digital phase contrast images were captured by high content analysis system-operetta CLS^TM^. Scale bar: 100 µm and 50 µm (for enlarged images). Figure S9: In vitro cytotoxicity of **TTCI** prodrugs (A) and **TTCI** NPs (B) at various concentrations against BxPC3 cells after 48 h incubation. Digital phase contrast images of living BxPC3 cells were captured by high content analysis system-operetta CLS^TM^ treated with **TTCI** prodrugs and **TTCI** NPs at various concentrations.
