# Supplementary material for: Smart Design of Mitochondria-Targeted and ROS-Responsive CPI-613 Delivery Nanoplatform for Bioenergetic Pancreatic Cancer Therapy
Source: Nanomaterials (Basel). 2021 Oct 28;11(11):2875. doi: 10.3390/nano11112875 (PMC8617807; doi:10.3390/nano11112875)
Supplement: Supplementary file 1 [file nanomaterials-11-02875-s001.zip › nanomaterials-1388820-supplementary.pdf]

nanomaterials

MDPI

Supplementary Materials

Smart Design of Mitochondria-Targeted and ROS-Responsive CPI-613 Delivery Nanoplatform for Bioenergetic Pancreatic Cancer Therapy

Yi-Mei Zhang [1,2], Meng Xia [1], Rui Ao [1], Li-Xia Gao [1], Yan Tang [1], Jiu-Hong Huang [1], Ya-Fei Luo [1], Zhong-Zhu Chen [1], Bo-Chu Wang [2] and Zheng Huang [1,*]

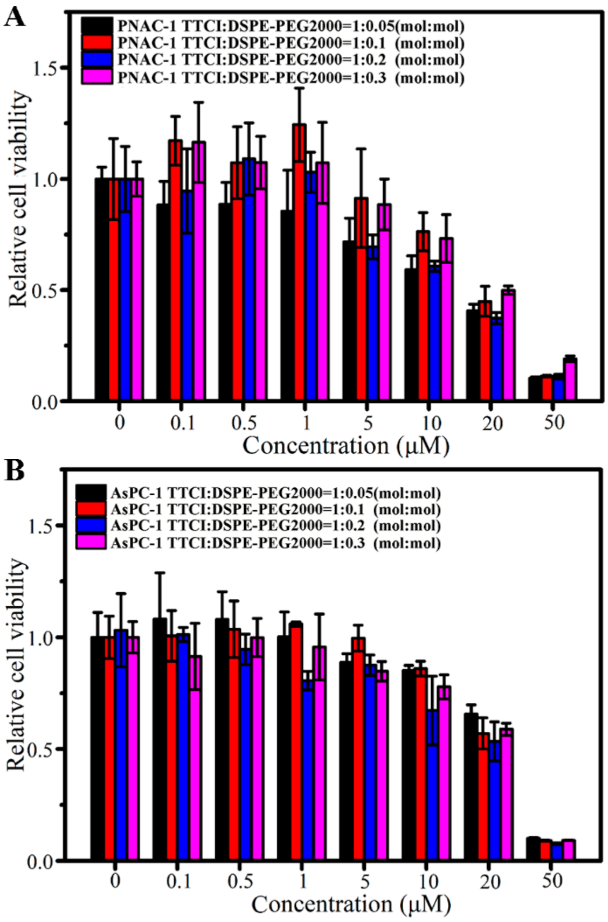

Figure S1. In vitro cytotoxicity of **TTCI** NPs constructed with different molar ratios of **TTCI**:DSPE-PEG2000 in PNAC-1 (**A**) and AsPC-1 (**B**) cells treated with various concentrations for 48 h. Data represent mean ± SD (n = 3).

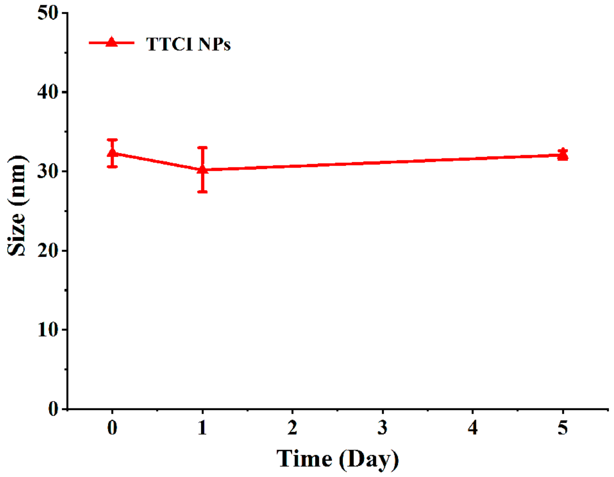

Figure S2. Mean particle sizes of **TTCI** NPs constructed with **TTCI**:DSPE-PEG2000 at a molar ratio of 1:0.2 at different time points (DLS at room temperature). Data represent mean ± SD (n = 3).

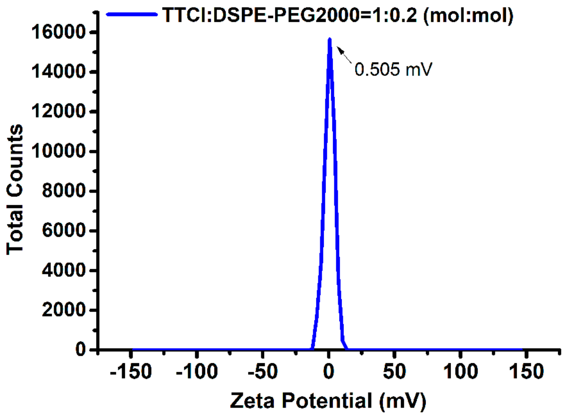

Figure S3. Zeta-potentials of **TTCI** NPs constructed with **TTCI**:DSPE-PEG2000 at a molar ratio of 1:0.2 (DLS at room temperature).

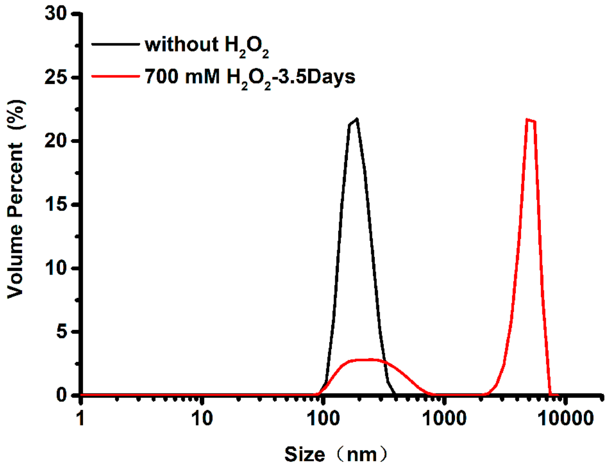

Figure S4. Size distribution of **TTCh** NPs after treatment with 700 mM H_2O_2 for different time.

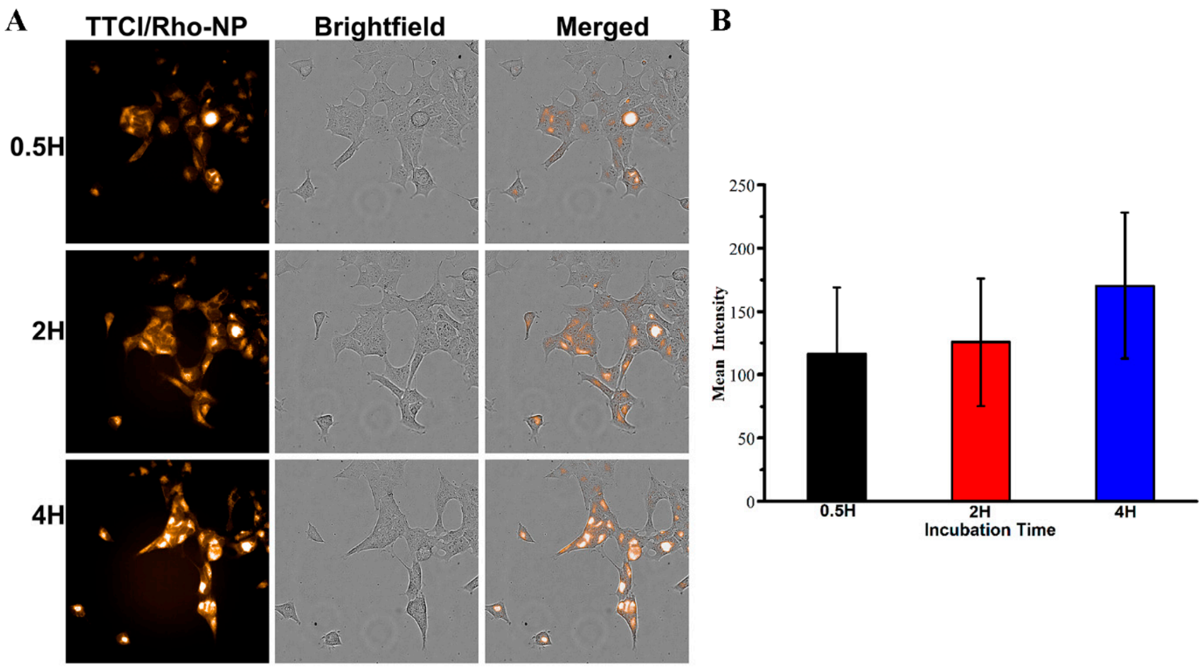

Figure S5. Images of the BxPC-3 cells treated with Rhodamine B-loaded **TTCI/Rho** NPs at the concentration of 20 μM for 0.5, 2, 4 h, respectively. For each row, left: **TTCI/Rho** NPs; middle: bright field; right: merged image. The images were captured by high content analysis system-operetta CLS™ (**A**). Scale bars: 50 μm. the mean intensity of rhodamine fluorescent in the images at different incubation times calculated by the imageJ software (**B**).

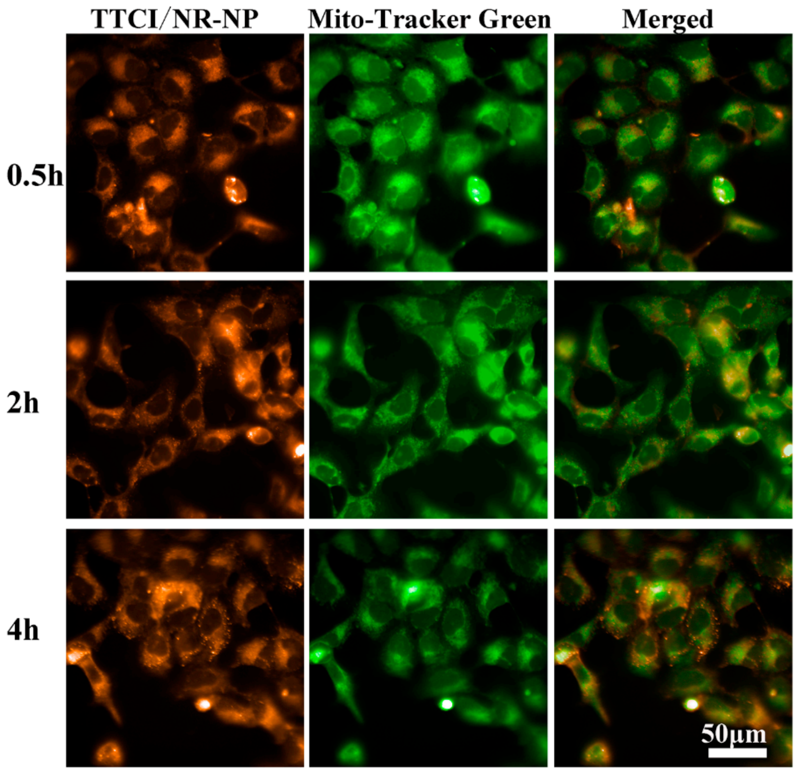

Figure S6. Mitochondria-targeting ability of **TTCI/NR** NPs in vitro: Images of the BxPC3 cells treated with **TTCI/NR** NPs at the concentration of 20 μM for 0.5, 2 and 4 h captured by high content analysis system-operetta CLS™. For each row, from left to right: **TTCI/NR** NPs; mitochondria stained by MitoTracker Green; merged image. Scale bars: 50 μm.

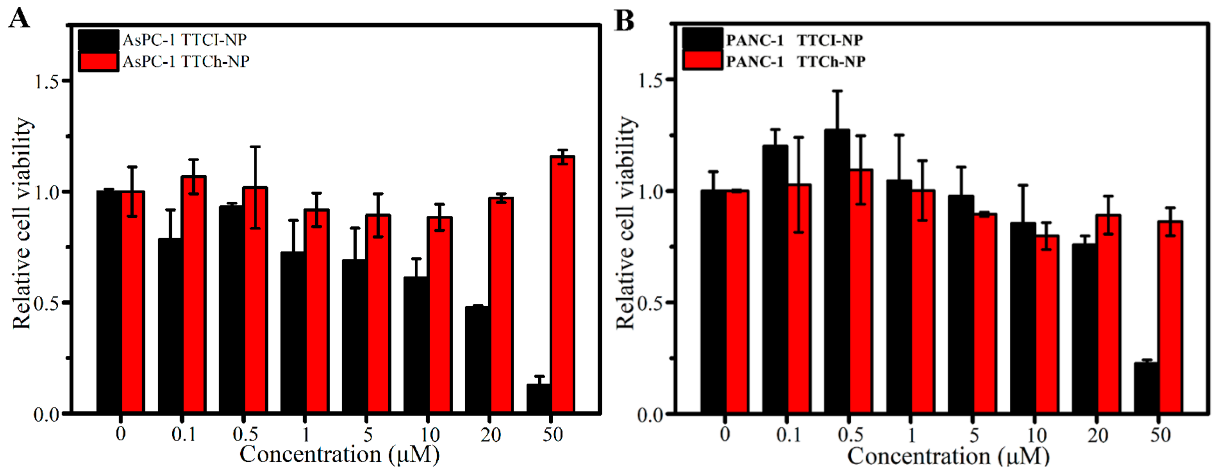

Figure S7. Cell viability of AsPC-1 (**A**) and PANC-1 (**B**) cells after being treated with different concentration of **TTCI** NPs and **TTCh** NPs for 48 h. Data represent mean ± SD (n = 3).

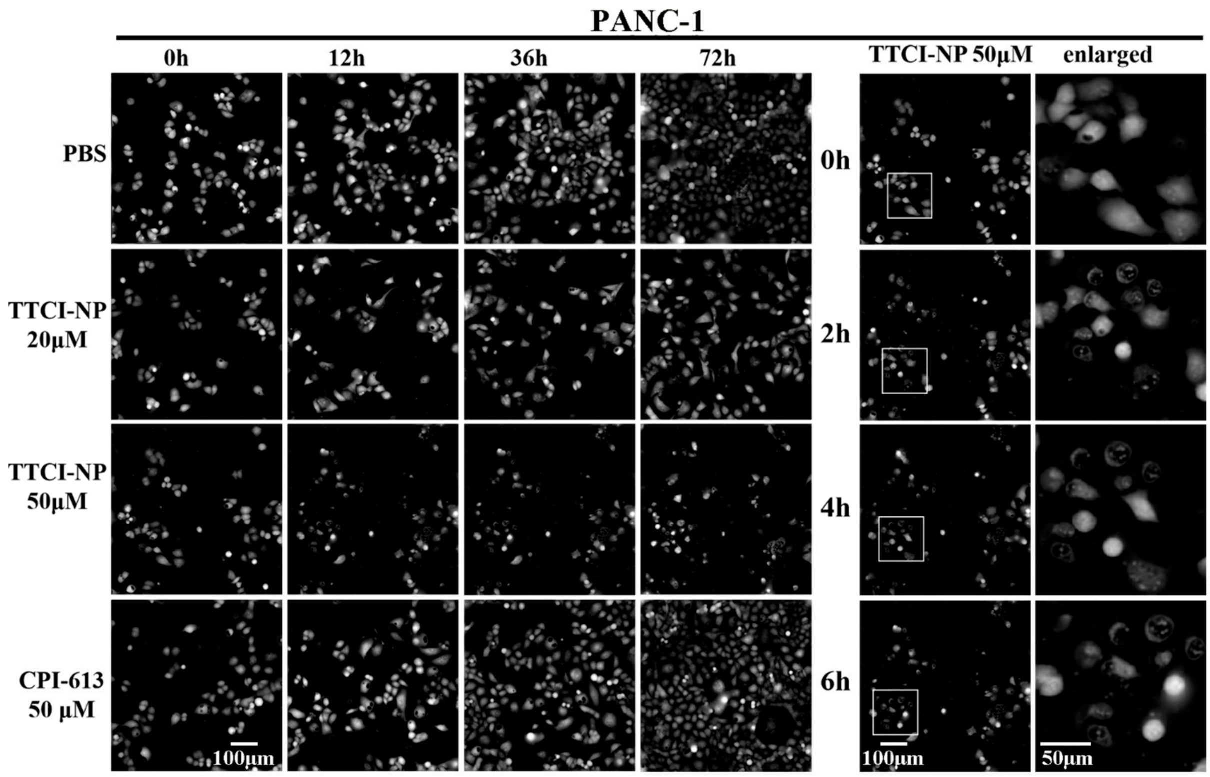

Figure S8. Digital phase contrast images of living PANC-1 cells treated with PBS, 20 μM **TTCI** NPs, 50 μM **TTCI** NPs and 50 μM CPI-613 at different times. Digital phase contrast images were captured by high content analysis system-operetta CLSTM. Scale bar: 100 μm and 50 μm (for enlarged images).

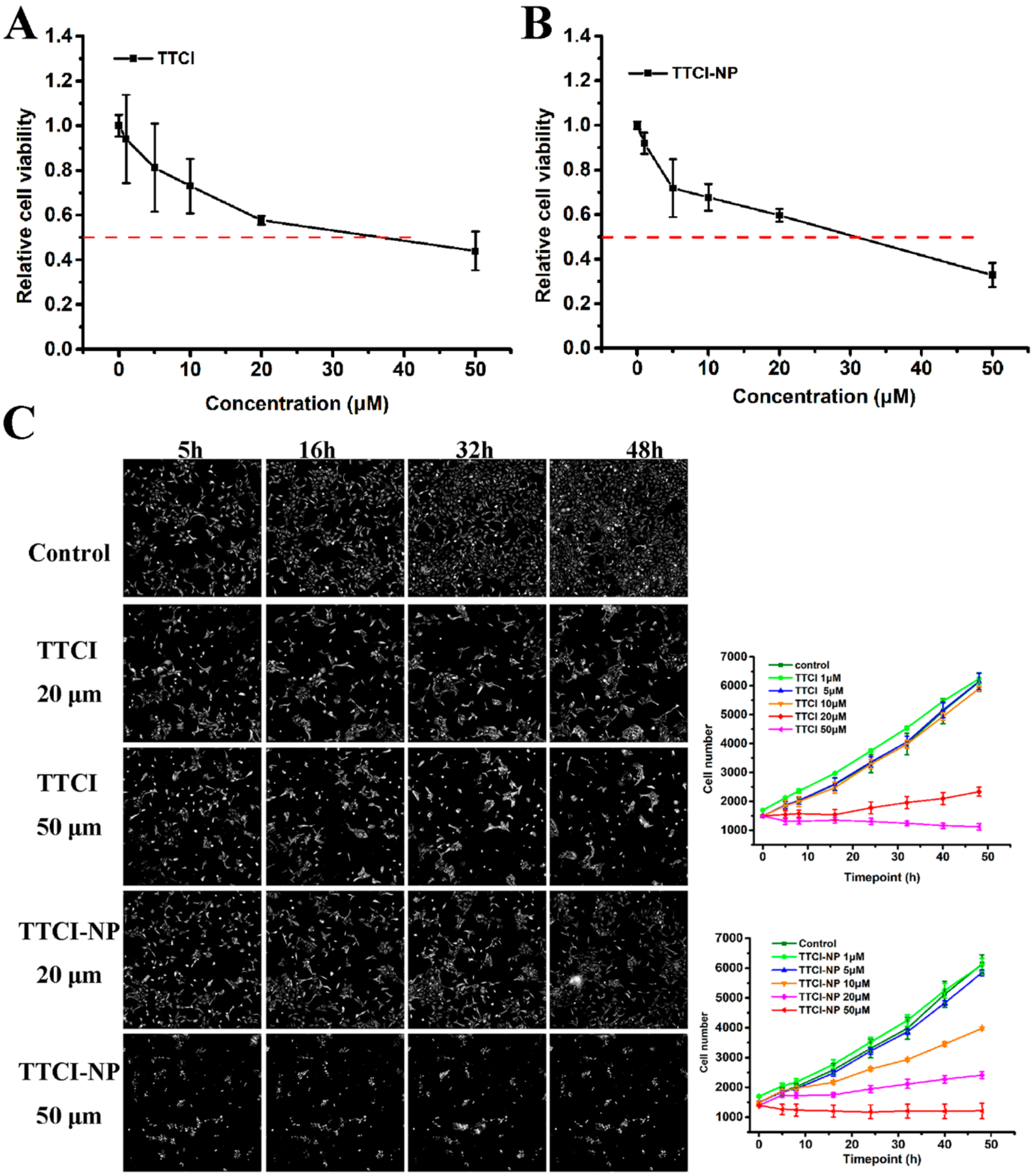

Figure S9. In vitro cytotoxicity of **TTCI** prodrugs (**A**) and **TTCI** NPs (**B**) at various concentrations against BxPC3 cells after 48 h incubation. Digital phase contrast images of living BxPC3 cells were captured by high content analysis system-operetta CLS™ treated with **TTCI** prodrugs and **TTCI** NPs at various concentrations (**C**).
